# Local Overheating of Biotissue Labeled With Upconversion Nanoparticles Under Yb^3+^ Resonance Excitation

**DOI:** 10.3389/fchem.2020.00295

**Published:** 2020-05-08

**Authors:** Ivan V. Krylov, Roman A. Akasov, Vasilina V. Rocheva, Natalya V. Sholina, Dmitry A. Khochenkov, Andrey V. Nechaev, Nataliya V. Melnikova, Alexey A. Dmitriev, Andrey V. Ivanov, Alla N. Generalova, Evgeny V. Khaydukov

**Affiliations:** ^1^Scientific Research Center “Crystallography and Photonics,” Russian Academy of Sciences, Moscow, Russia; ^2^Center of Biomedical Engineering, Institute of Molecular Medicine, Sechenov University, Moscow, Russia; ^3^National Medical Research Center for Oncology, Ministry of Health of Russian Federation, Moscow, Russia; ^4^Medicinal Chemistry Center, Togliatti State University, Togliatti, Russia; ^5^Institute of Fine Chemical Technologies, Moscow Technological University, Moscow, Russia; ^6^Engelhardt Institute of Molecular Biology, Russian Academy of Sciences, Moscow, Russia; ^7^Laboratory of Polymers for Biology, Shemyakin-Ovchinnikov Institute of Bioorganic Chemistry, Russian Academy of Sciences, Moscow, Russia

**Keywords:** upconversion nanoparticles, bioimaging, photothermal material, hyperthermia, local overheating, near-infrared irradiation, heat shock proteins, biotissue laser heating

## Abstract

Local overheating of biotissue is a critical step for biomedical applications, such as photothermal therapy, enhancement of vascular permeability, remote control of drug release, and so on. Overheating of biological tissue when exposed to light is usually realized by utilizing the materials with a high-absorption cross section (gold, silica, carbon nanoparticles, etc.). Here, we demonstrate core/shell NaYF_4_:Yb^3+^, Tm^3+^/NaYF_4_ upconversion nanoparticles (UCNPs) commonly used for bioimaging as promising near-infrared (NIR) absorbers for local overheating of biotissue. We assume that achievable temperature of tissue labeled with nanoparticles is high enough because of Yb^3+^ resonance absorption of NIR radiation, whereas the use of auxiliary light-absorbing materials or shells is optional for photothermal therapy. For this purpose, a computational model of tissue heating based on the energy balance equations was developed and verified with the experimentally obtained thermal-graphic maps of a mouse in response to the 975-nm laser irradiation. Labeling of biotissue with UCNPs was found to increase the local temperature up to 2°C compared to that of the non-labeled area under the laser intensity lower than 1 W/cm^2^. The cellular response to the UCNP-initiated hyperthermia at subcritical ablation temperatures (lower than 42°C) was demonstrated by measuring the heat shock protein overexpression. This indicates that the absorption cross section of Yb^3+^ in UCNPs is relatively large, and microscopic temperature of nanoparticles exceeds the integral tissue temperature. In summary, a new approach based on the use of UCNP without any additional NIR absorbers was used to demonstrate a simple approach in the development of photoluminescent probes for simultaneous bioimaging and local hyperthermia.

**Graphical Abstract:**
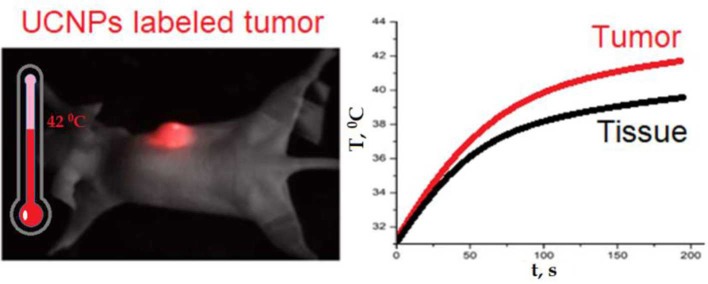
Overheating of UCNP-labeled tumor.

## Introduction

Recently, upconversion nanoparticles (UCNPs) and UCNP-based nanoconstructions showed a great potential in biomedical applications for near-infrared (NIR)–to–NIR bioimaging (Chen et al., 2014; Wu et al., 2014; Li et al., 2015; Generalova et al., 2016), photodynamic (Khaydukov et al., 2016; Xu et al., 2017; Qiu et al., 2018; Liu et al., 2019), and photothermal therapy (PTT) (Zhu et al., 2016; Chan et al., 2018). Upconversion nanoparticles (NPs) consist of inorganic host matrix, typically NaYF_4_, codoped with Yb^3+^, Er^3+^ or Yb^3+^, Tm^3+^ lanthanide ions. The UCNP luminescence and excitation lines are located in the so-called “biological transparency window,” whereas the anti-Stokes luminescence signal shift allows the absence of autofluorescence. Unique UCNP properties provide NIR-to-visible and NIR-to-NIR luminescence suitable for bioimaging (Kobayashi et al., 2009), long-term visualization implemented even at the level of single NP (Liu et al., 2018), and the excellent excitation depth in biological tissue of up to 1 cm (Zhan et al., 2013). In addition, UCNPs are characterized by superior photostability, non-blinking (Wu et al., 2009), long lifetimes (Zhan et al., 2013; Pilch et al., 2017), low cytotoxicity (Guller et al., 2015, 2018; Vedunova et al., 2016), and high spatial resolution during bioimaging (Zhan et al., 2011; Chen et al., 2012; Xu et al., 2013; Khaydukov et al., 2014; González-Béjar et al., 2016; Generalova et al., 2017).

Nanoparticles have great potential for PTT. By providing heat at the nanoscale, they facilitate therapeutic efficacy and reduce side effects for normal tissue compared to conventional ultrasound and microwave methods. Heat generation from the NP consists of the absorption of incident photons, conversion of light energy into heat, and its transfer from the NP to the biotissue. Typically, the photothermal properties of UCNP nanoconstructions are achieved by shell formation or inclusion of NPs with a high-absorption cross section. For example, temperature feedback of UCNPs coated with carbon layer as a light absorber was used for the precise tumor PTT with minimal damage to normal tissue (Zhu et al., 2016). To enhance the absorption of plasmon resonance, a more complex double-coated UCNP structure with layers of iron and gold was proposed (Cheng et al., 2011). Graphene- and silica-based UCNP complexes for local overheating were also demonstrated (Liu et al., 2013; Gulzar et al., 2018). In addition, the specific optical properties of UCNPs are ideal for real-time luminescent temperature measurements in the physiological range (Sedlmeier et al., 2012; Li et al., 2017). However, the UCNP structure with additional layers or NPs as light-absorbing materials for PTT is often cumbersome and complicates the preparation of nanoprobes.

Magnetic NPs are often used as a model of NP-mediated hyperthermia. This approach was first proposed by Gilchrist et al. (1957), who introduced the concept of injecting magnetic NPs (20–100 nm) into lymphatic channels to heat residual cancer cells under an alternating magnetic field (AMF). Because delivering magnetic NPs via direct injection into the tumor could result in much more effective and selective heating when compared to other heating techniques, this approach was discussed as more safe and clinically promising (Jordan et al., 2009). Since then, significant efforts have been directed to the development of novel magnetic NPs, including targeted ones, and clinical AMF systems, resulting in clinical trials and industrial solutions (Cruz et al., 2017; Chang et al., 2018). Thus, within the past two decades, phases 1 and 2 of clinical studies of intratumorally delivered magnetic NPs treated by AMF were successfully conducted for patients with glioblastoma and prostate cancers (Johannsen et al., 2010; Maier-Hauff et al., 2011). We believe that UCNPs, which are comparatively new in medicine biotechnology, could be proposed as a promising alternative to magnetic NPs in terms of NP-mediated hyperthermia induction.

Herein, we synthesized core-shell UCNPs NaYF_4_:Yb^3+^:Tm^3+^/NaYF_4_ modified with a PEG shell aiming to demonstrate the possibility of PTT based on resonant excitation ^2^F_5/2_ → ^2^F_7/2_ of Yb^3+^ at low apparent absorption cross section. Our results indicate that Yb^3+^ absorption cross section in NPs is rather sufficient to increase the local temperature of the biological tissue to 2°C at a 975-nm radiation with intensity of <1 W/cm^2^. Thus, theranostic NPs for simultaneous bioimaging and local hyperthermia can be easily designed because of their own absorption of UCNPs without impregnation with adjuvant light absorbers.

## Results and Discussion

**Figure 1:**
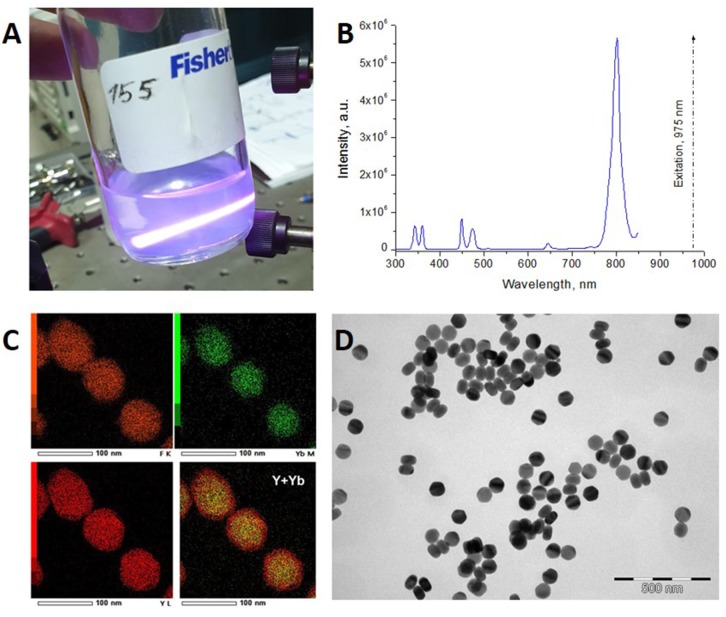
**(A)** UCNPs colloid illuminated with a 975-nm laser beam. Blue traces of photoluminescence illustrate conversion of NIR radiation in NPs. The image was taken via the interference filter Semrock (842-nm blocking edge BrightLine® short-pass filter Semrock, New York, NY, USA) cutting off 15 W/cm^2^ excitation at 975 nm. **(B)** Photoluminescence spectrum of UCNPs under excitation at 975 nm. **(C)** Elemental EDX mapping of NPs, overlay Y (red), and Yb (green) indicates core/shell structure of UCNPs. **(D)** Transmission electron microscopy (TEM) images of as-synthesized core/shell NaYF_4_:Yb^3+^, Tm^3+^/NaYF_4_ UCNPs with hexagonal prism shape (75-nm base and 50-nm height).

In order to demonstrate simultaneous bioimaging and local hyperthermia, we synthesized UCNPs with a core/shell structure. The UCNP is an inorganic crystal matrix of the most popular composition NaYF_4_ codoped with ytterbium (Yb^3+^) and thulium (Tm^3+^) ions with an undoped shell NaYF_4_. The host matrix material should be optically transparent for light excitation and emission and have low phonon energy for efficient energy transfer inside the particle (Chen et al., 2014; Wu et al., 2014). In this study, we proposed that UCNPs consist of the hexagonal β-phase of NaYF_4_ and two types of ions: a sensitizer (Yb^3+^) and an activator (Tm^3+^) (Figure 1). Yb^3+^ is capable of absorbing laser excitation at 975 nm and non-radiatively transfers this energy to Tm^3+^ ions. Tm^3+^ have multiple metastable excited states with long lifetimes (sub-ms) (Auzel, 2004). Energy transfer from Yb^3+^ to Tm^3+^ can occur several times. Eventually, Tm^3+^ has several emission lines from 800 to 345 nm (Figure 1b).

The as-synthesized hydrophobic UCNPs were hydrophilized using amphiphilic poly(maleic anhydrate-*alt-1-*octadecene) followed by surface modification using cross-linker poly(ethylene glycol) diglycidyl ether. This approach led to the formation of a “corona”-like negatively charged (ξ-potential was −41,1 mV) structure with PEG molecules on the UCNP surface. The PEG layer determines biocompatibility, non-cytotoxicity, and low blood protein adsorption of UCNPs (Generalova et al., 2016). The cross-linked shell on UCNP surface (PEG-UCNPs) maintained the colloidal stability for at least a month and was not affected by electrolytes (0.15 M NaCl and buffers). It should be noted that PEGylation of UCNPs is one of the most common methods to increase the biocompatibility of NPs, prolong circulation time, and, as a result, to increase passive accumulation in tumors due the enhanced permeability and retention effect (Generalova et al., 2016; Han et al., 2018).

**Figure 2:**
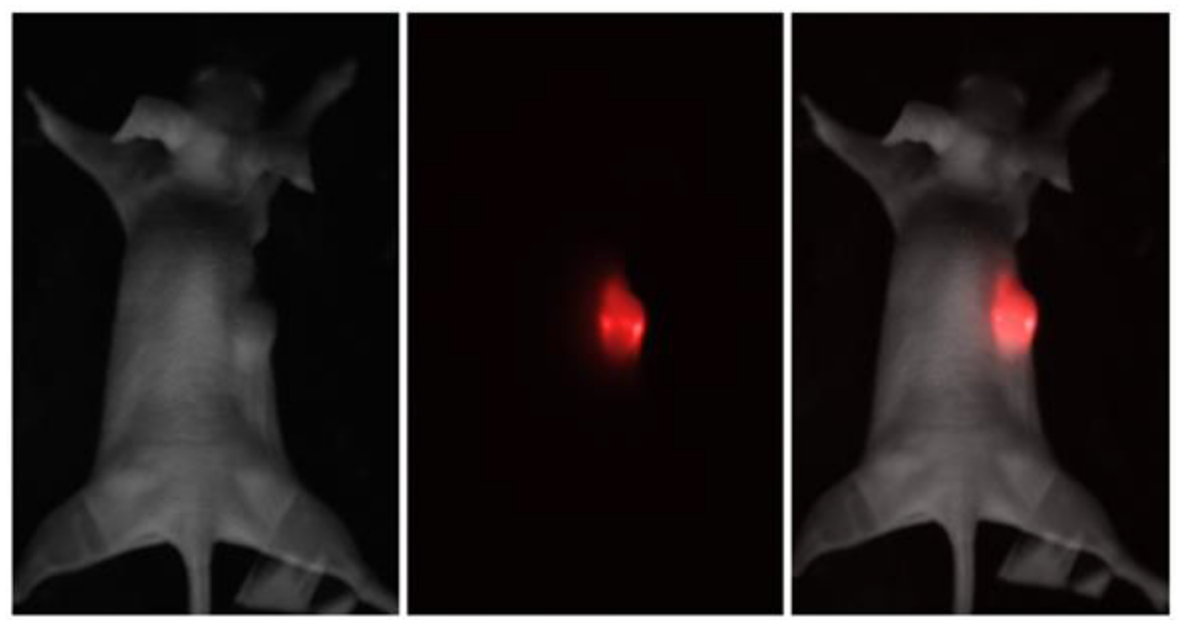
Brightfield, epiluminescent, and overlay images of the immunodeficient mouse, bearing a grafted subcutaneous A375 tumor. The UCNP luminescence at 800 nm is represented in red color.

Melanoma xenograft-bearing mice were used to demonstrate self-temperature effects of UCNPs for theranostic applications. For this purpose, 2 × 10^6^ human melanoma A375 cells were subcutaneously inoculated into immunodeficient Balb/c nu/nu mice (see Methods section for details) and were grown for 15 days until the tumors reached 150 ± 20 mm^3^ in volume, as shown in Figure 2. Then, 100-μL phosphate-buffered saline (PBS) solution containing 200 μg PEG-UCNPs was injected into the tumor-surrounding tissues and incubated for 2 h. In order to visualize the UCNPs in tumor, we performed an *in vivo* whole-animal imaging using custom-developed epiluminescence imaging system. The NIR-to-NIR system realizes raster scanning excitation at 975 nm with wide-field EMCCD detection at 800 nm. A strong luminescence signal was detected in the tumor, while normal tissues remained unlabeled (Figure 2).

**Figure 3:**
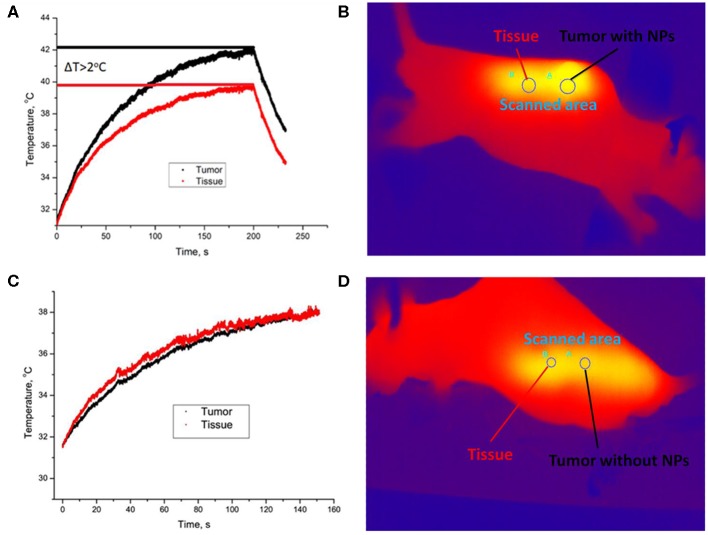
Temperature image of immunodeficient mice bearing a grafted A375 tumor during 975-nm laser irradiation at the intensity of 1 W/cm^2^. Melanoma xenograft labeled with UCNPs **(A)** and control (no UCNPs) **(B)**. Area A is for tumor, and area B is for normal tissue. A temperature increase during laser treatment in the UCNP-labeled **(C)** and control **(D)** mice. The tumor (area A) is shown with a black curve, and normal tissue (area B) is visualized with a red curve.

After imaging the tumor, the *in vivo* heating procedure was started. Heating was provided by 975-nm laser beam scanning with an adjusted area due to a system of galvanic mirrors. A375 xenograft-bearing mouse was fixed to the plate with a duct tape. The mouse temperature was monitored by a Gobi-384-GigE-7098 Camera (Leuven, Belgium). The temperature of UCNP-labeled tumor was found to be higher than temperature of the surrounding tissue (Figures 3A,B). After 3 min of laser treatment, the detected temperature gap was ~2°C. In order to prove that tumor overheating is associated with UCNPs impregnated in the tumor, a control group of unlabeled A375 melanoma xenografts was used. Surprisingly, the effect of overheating was not observed (Figures 3C,D). Note that the ablation temperature of biotissue is usually discussed as 42°C or higher (Zhu et al., 2016). In our experiments, this temperature was reached during 3 min of laser treatment, while the temperature of the treated normal tissue was in a safe range.

We have developed a computational model that predicts the heating of biotissue with variable parameters, namely, laser treatment time, NP concentration, and laser beam intensity.

**Figure 4:**
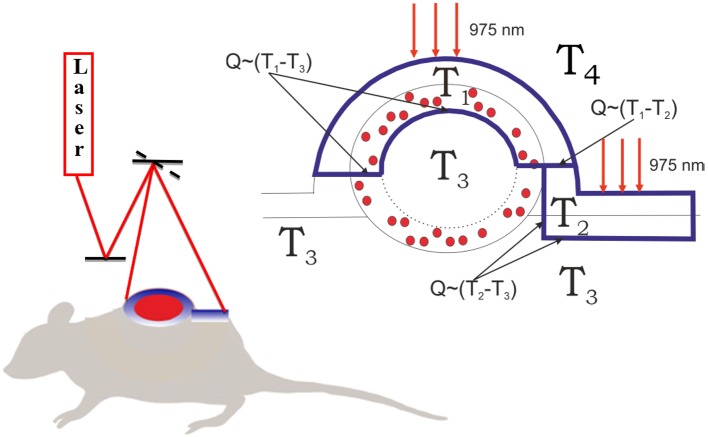
Schematics of biotissue and tumor temperature modeling. The outer layer of the tumor tissue is labeled with UCNPs (red dots). The tumor and normal tissue were irradiated with 975-nm laser excitation. The laser dose was evenly released due to a galvanic mirror scanning system. The heated fields are marked with a blue line. *T*_3_ is the temperature in the tumor core equal to the temperature of the non-irradiated tissue, *T*_2_ is the temperature of the irradiated skin layer, *T*_1_ is the temperature of the irradiated interstitial tumor tissue, and *T*_4_ is the environment temperature. *T*_1_ ≥ *T*_2_ ≥ *T*_3_ ≥ *T*_4_.

For this purpose, we modified a temperature- and time-dependent model described earlier (Zhu et al., 2016). We proposed four temperature fields in the system “mouse-air” (Figure 4). In details, *T*_1_ is the temperature of the tumor layer impregnated by UCNPs and irradiated by a 975-nm laser beam. *T*_2_ corresponds to the temperature of the UCNP-free biotissue layer also irradiated with the laser beam. *T*_3_ is the average temperature of the whole body, and *T*_4_ is the environment temperature. The main model assumptions are as follows:

1. *Q*_*i* → *j*_ ~ (*T*_*i*_ − *T*_*j*_), where *Q*_*i*−>*j*_ is the energy passing between *i*_th_ and *j*_th_ areas.
2. There is the same temperature *T*_*i*_ for a whole *i*_th_ area.
3. The absorber (NP) distribution is uniform.

A solid tumor is known to have the specific pressure distribution (Liu et al., 2016). Typically, the solid tumor divides into three regions: (1) the central region that contains the necrotic core; (2) the tumor periphery region with abundant blood vessels, which is the main source of tumor interstitial fluid; and (3) the intermediate region, where the increased core pressure equalizes with normal tissue fluid pressure (Liu et al., 2011). As a result, the injected NPs are redistributed into the tumor and form a specific surface layer impregnated with UCNPs. Based on our previous results (Generalova et al., 2016), we estimate the thickness of the layer with UCNPs as ~2 mm. It should be noted that our estimation correlates with the data obtained in Liu et al. (2016). The energy balance equation in the first area can be written as follows:

$$\begin{array}{r} Cm_{1}\frac{\text{d}T_{1}}{\text{d}t}=Q_{in}-\;Q_{out}=Q_{1}-\left(Q_{1\to 2}\;+Q_{1\to 3}\;+Q_{1\to 4}\right)\\ =\;N\sigma I+\;P\left(1-e^{-kd}\right)-\;h_{12}S_{12}\left(T_{1}\left(t\right)-T_{2}\left(t\right)\right)\\ -h_{13}S_{13}\left(T_{1}\left(t\right)-\;T_{3}\left(t\right)\right)\;-h_{14}S_{14}\left(T_{1}\left(t\right)-\;T_{4}\right), \end{array}$$

where *C* is the specific heat, *m*_1_ is the mass of the surface layer, *T*_*j*_ is the temperature in the *j*-th area, *t* is time, *N* is the number of ytterbium ions absorbing laser radiation, σ is the absorption cross section of ytterbium, *I* is the intensity of the incident laser radiation, *P* is the power of the laser radiation, *k* is the absorption coefficient, *S*_*ij*_ is the heat exchange surface area between the *i*-th and *j*-th areas, and *h*_*ij*_ is the heat transfer coefficient.

Simplifying, we obtain

$$\begin{array}{l} \frac{dT_{1}}{dt}=\;A_{1}-B_{12}\left(T_{1}\left(t\right)-T_{2}\left(t\right)\right)-B_{13}\left(T_{1}\left(t\right)-T_{3}\left(t\right)\right)\\ -B_{14}\left(T_{1}\left(t\right)-T_{4}\right),\\ A_{1}=\frac{N\sigma I+\;P\left(1-e^{-kd}\right)}{Cm_{1}},B_{ij}=\;\frac{h_{ij}S_{ij}}{Cm_{1}}. \end{array}$$

Writing the similar energy balance equations in second and third areas, we obtain the following system:

$$\begin{array}{l} \frac{dT_{1}}{dt}=\;A_{1}-B_{12}\left(T_{1}\left(t\right)-T_{2}\left(t\right)\right)-B_{13}\left(T_{1}\left(t\right)-T_{3}\left(t\right)\right)\\ -\;B_{14}\left(T_{1}\left(t\right)-T_{4}\right)\\ \frac{dT_{2}}{dt}=\;A_{2}+B_{21}\left(T_{1}\left(t\right)-T_{2}\left(t\right)\right)-B_{23}\left(T_{2}\left(t\right)-T_{3}\left(t\right)\right)\\ -\;B_{24}\left(T_{2}\left(t\right)-T_{4}\right)\\ \frac{dT_{3}}{dt}=A_{3}+B_{32}\left(T_{2}\left(t\right)-T_{3}\left(t\right)\right)+B_{31}\left(T_{1}\left(t\right)-T_{3}\left(t\right)\right)\\ -\;B_{34}\left(T_{3}\left(t\right)-T_{4}\right)\\ T_{1}\left(0\right)=T_{1}^{0}\\ T_{2}\left(0\right)=T_{2}^{0}\\ T_{3}\left(0\right)=T_{3}^{0} \end{array}$$

**Figure 5:**
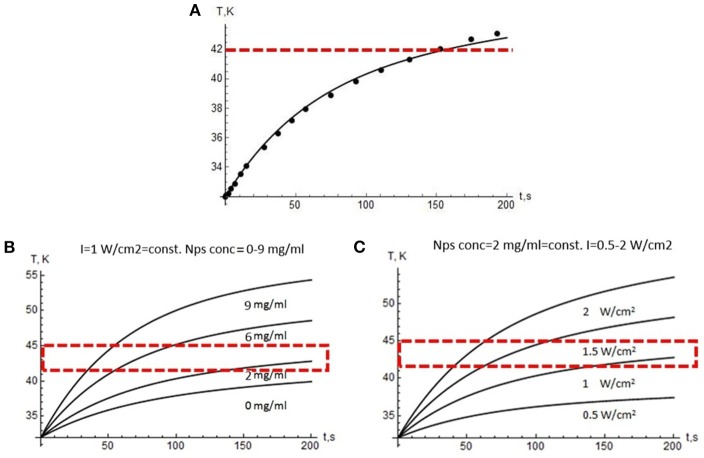
Modeling results. **(A)** Temperature growth during laser treatment in the tumor (dots—experiment, curve—modeling data). Red dotted line indicates the lower edge of the overheating region. Temperature profiles vs. 975 nm treatment time: **(B)** at a constant laser power density of 1 W/cm^2^ and the variable NP concentration, **(C)** at a constant NP concentration of 2 mg/mL and a variable power density from 0.5 to 2 W/cm^2^. Red dotted line indicates the range suitable for PTT.

This system was solved numerically, and the results are presented in Figure 5. The theoretical modeling allows predicting optimal physical parameters for overheating, taking into account the size and shape of the tumor. We have verified the theoretical model by comparing the experimental and calculated data (Figure 5A). Figures 5B,C show calculated laser intensities and NP concentrations with allowance for the need to maintain the temperature in the range from 42°C to 45°C on the plateau because the exposure time in potential medical use is quite long.

**Figure 6:**
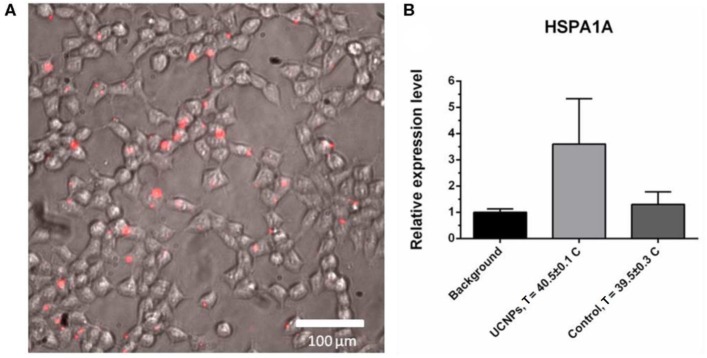
Upconversion nanoparticle (in red) accumulation in A375 cells, 30-min incubation with 50 μg/mL UCNPs **(A)**. The *HSPA1A* expression in A375 human melanoma cells, quantitative PCR (qPCR) assay: background sample (no UCNPs, no NIR), treated sample (0.1 mg/mL UCNPs, NIR irradiation), control sample (no UCNPs, NIR irradiation). The *HSPA1A* expression in each sample was normalized to that in the background sample **(B)**.

We provide *in vitro* experiment on the heat shock protein (HSP) expression in response to overheating associated with UCNPs upon 975-nm irradiation. Overheating in biotissue causes several hazardous effects that lead to cell death through necrosis (proinflammatory response) or apoptosis, which is more attractive because apoptosis inhibits the inflammatory response (Melamed et al., 2015). Heat shock proteins are a family of proteins involved in the folding and maturation of proteins, and they have been identified as key determinants of cell survival under stressful conditions (Kennedy et al., 2014; Wu et al., 2017), and, in particular, in the apoptotic process (Lanneau et al., 2008). Here, we evaluated the expression of HSP70 family protein as a marker for the cellular response to overheating caused by UCNP photoactivation. For this purpose, cells were incubated with 0.1 mg/mL UCNPs, and rapid (within 30 min) accumulation of UCNPs in the cells was demonstrated, confirming high biocompatibility (Figure 6A). The cells were then irradiated until a critical temperature was reached for HSP expression. Although the temperature of the treated sample was 40.5°C ± 0.1°C that is lower than 41°C to 42°C usually required for HSP induction, the relative expression level of the *HSPA1A* gene (HSP70 family) was approximately 3.5 times higher compared to the untreated sample (Figure 6B). In contrast, there was no significant increase in *HSPA1A* gene expression for cells heated at the same laser settings and at a similar final temperature (39.5°C ± 0.3°C). Because this could be explained by the temperature gap between the UCNP-loaded sample and the UCNP-free sample, we performed an additional experiment with equal temperature for both samples (40.6°C ± 0.5°C). We found an increase in the relative level of *HSPA1A* expression for the UCNP-loaded sample that was about 4.5 times higher compared to the non-treated sample and no increase for the control heating without the UCNP. We suppose that this could be explained with the localized nature of UCNP-induced heating. In other words, the NP temperature can be significantly higher than the temperature of the medium. This effect was previously demonstrated for iron oxide NPs when particle-based hyperthermia has increased cytotoxicity compared to conventional hyperthermia in a water bath at the same measured heat dose (Ogden et al., 2009). Previously, it was also demonstrated that the exposure of A375 melanoma cells to temperatures below 43°C does not significantly affect viability levels (Mantso et al., 2018). Therefore, thermal damage caused by particles can be discussed as more effective than conventional integrated heating. It should be also noted that some metal-based NPs can themselves induce HSP expression through oxidative stress (Ahamed et al., 2010; Vecchio et al., 2012). However, in this study, the addition of UCNPs without NIR irradiation did not lead to significant HSP induction (Supplementary Data 1).

In order to explain the observed experimental facts and that theory converges with experimental data, we estimated the absorption cross section of ^2^F_5/2_ → ^2^F_7/2_ transition for Yb^3+^ ions in NPs, as 2 × 10^−19^ cm^2^. At present, a relatively low value of 10^−20^ cm^2^ as determined at 980 nm in the germanosilicate glass matrix (Paschotta et al., 1997) without allowance for the crystal matrix symmetry is widely accepted as the reference for the absorption cross section of Yb^3+^ in the literature. It is well-known that σ_abs_ can vary depending on the type of the crystalline matrix due to splitting Stark levels. For example, the effective energy absorption of Nd^3+^, which is also used for optical imaging, can vary by three orders of magnitude (Kaminskii et al., 1980, 1982a,b). Fluoride materials with low symmetry, for example, LiLnF_4_ and BaLn_2_F_8_ crystals, were shown to increase the absorption cross section by two orders of magnitude compared to oxide matrices with a cubic structure (Brown et al., 1969; Caspers and Rast, 1975; Esterowitz et al., 1979a,b; Da Gama et al., 1981).

Moreover, the question about the absorption cross section for doping ions in nanocrystals is probably even more complicated because the crystal symmetry at the nanoscale level can be violated because of geometric factors. We believe that the value of Yb^3+^ absorption cross section is an open question today and requires a detailed analysis. Further experiments are needed, although our data indicate the absorption cross section of Yb^3+^ in the β-NaYF_4_ matrix is relatively large.

## Conclusions

We demonstrated core/shell NaYF_4_: Yb^3+^, Tm^3+^/NaYF_4_ UCNPs as promising NIR absorbers for local biotissue overheating. The critical photothermal temperature (≥42°C) was achieved due to Yb^3+^ absorption of NIR radiation without the assistant light-absorbing materials. A computational model of tissue heating based on the energy balance equations was developed and verified with the experimentally obtained thermal-graphic maps of the mouse in response to the 975-nm laser exposure. Our modeling results indicate that the absorption cross section of Yb^3+^ in UCNPs is larger than the literature value, while microscopic temperature of NPs exceeds the integral tissue temperature. In summary, a novel approach based on UCNP application without additional NIR absorbers was developed and applied to demonstrate the possibility of these photoluminescent probes to be used for simultaneous bioimaging and local hyperthermia. We assume that demonstrated temperature modality of UCNPs will be perspective as a new strategy for combined cancer therapy.

## Methods

### UCNP Synthesis

The synthesis of lanthanide-doped UCNPs based on NaYF_4_ matrix was performed as described earlier (Grebenik et al., 2013; Generalova et al., 2016). Briefly, the synthesis is based on the coordinate stabilization of yttrium, ytterbium, and thulium metal salts in a solution of oleic acid and octadecene. It was carried out under heating in an oxygen-free atmosphere. The product of synthesis is hydrophobic monodisperse NPs (75 ± 5 nm) (Supplementary Data 2) with a core@shell structure (β-NaYF_4_: 18% Yb^3+^, 0.6% Tm^3+^@NaYF_4_), which form stable colloids in non-polar organic solvents, such as hexane and chloroform.

### Intercalation of UCNPs Using Amphiphilic Polymer Followed by Cross-Linking With PEG-Diglycidyl Ether

The coating of UCNPs with an amphiphilic polymer poly(maleic anhydrate-*alt-1-*octadecene) (PMAO) by solvent evaporation method was performed according to the previously described protocol (Guller et al., 2015). Resulted aqueous dispersion of UCNPs-PMAO containing 0.8 mg of UCNPs was centrifuged at 13,400 rpm for 10 min. The pellet was dispersed in 1 mL PBS buffer (pH 7.0), and 40 μL 1.5% PEG-diglycidyl ether aqueous solution was added. The mixture was vortexed, sonicated for 5 min, and stirred for 1 h at 95°C. Thereafter, the mixture was centrifuged again at 13,400 rpm for 10 min with a PBS buffer addition (this procedure was repeated three times to remove an unreacted cross-linker), and dispersed in 1 mL of PBS buffer (pH 7.0). For detail see Generalova et al. (2016).

### Transmission Electron Microscopy (“TEM”)

High-angle annular dark field (HAADF) scanning TEM and high-resolution TEM studies were performed using a JEOL ARM200F cold FEG double aberration corrected electron microscope operated at 200 kV and equipped with a large solid-angle CENTURIO EDX detector and Quantum EELS spectrometer (JEOL, Tokyo, Japan for all equipment). A TEM sample was prepared by sample dissolution in methanol and deposition on hollow carbon Cu grid.

### Thermal Imaging Camera

The actual temperature of mice was measured by Xenics Gobi-384-GigE-7098 camera in real time. The working range of the camera is from 8 to 14 μm with an accuracy of 0.5°C and quantum yield 98% at the peak wavelengths 10 μm. Resolution of camera is 0.1 MPx, and the frame rate is 84 Hz. The data were transmitted by GigE-port to PC.

### Visualization System

The custom-developed epiluminescent optical imaging system was used to visualize the accumulation of UCNPs in the tumor. The mice were scanned by a laser beam with a diameter of 1 cm. The laser intensity at 975 nm was limited to 1 W/cm^2^. The detection of the photoluminescent signal was performed using a Falcon EMCCD camera (Raptor Photonics, Larne, Northern Ireland), equipped with the *F* = 0.95 objective. The interference filters (Semrock, New York, NY, USA) were placed in front of the lens, cutting the laser radiation. Exposure time of 2 seconds corresponded to two complete animal surface scans while taking the image by EMCCD camera.

### Cell Culture

Human melanoma A375 and human embryonic kidney HEK 293 cells were used in this research. All cells were grown in Dulbecco modified eagle medium (DMEM) growth medium supplemented with 10% fetal bovine serum (FBS), 2 μM l-glutamine, 100 μg/mL streptomycin, and 100 U/mL penicillin at 37°C in a 5% CO_2_ humidified atmosphere. The medium was replaced every 3 to 4 days.

### Cell Treatment

Cells were seeded in 48-well plate (50,000 cells per each well) in DMEM supplemented with 10% FBS and incubated overnight in CO_2_ incubator (37°C, 5% CO_2_). Then, the UCNPs were added to the cells to the final concentration of 0.1 mg/mL for 30 min. Then, the cells were heated using NIR irradiation (parameters). Cells without UCNPs and NIR irradiation were used as controls.

### RNA Isolation

RNA from A375 cells was isolated using Quick-RNA MiniPrep (Zymo Research, Irvine, CA, USA). RNA Lysis Buffer was added to the cells, and then RNA samples were purified with Zymo-Spin Columns according to the manufacturer's protocol. The RNA quality and concentration were evaluated on Agilent 2100 Bioanalyzer (Agilent Technologies, Santa Clara, CA, USA) and a Qubit 2.0 fluorometer (Thermo Fisher Scientific, Waltham, MA, USA).

### Reverse Transcription

One microgram of RNA from each sample was treated with DNase I (Thermo Fisher Scientific). Random hexamer primers (Evrogen, Moscow, Russia) were added to DNase treated RNA or no template control, incubated at 70°C for 5 min, and chilled on ice. Sixteen microliters of reaction mix for first-strand cDNA synthesis (1× reaction buffer and 1 mM dNTP mix; Evrogen) was added to each sample and incubated for 5 min at 25°C. Then, each sample was separated for two parts: one was used for reverse transcription and the other one for no-reverse-transcriptase control (RT-minus control). To generate the first-strand cDNA, 200 U of RevertAid Reverse Transcriptase (Thermo Fisher Scientific) was added to samples for reverse transcription, and then all samples were incubated for 10 min at 25°C followed by 60 min at 42°C. Before quantitative PCR, each sample was 10-fold diluted.

### Quantitative PCR

The qPCR was performed on 7500 Real-Time PCR System (Thermo Fisher Scientific) in 96-well plates. Reaction mix was prepared in 20 μL and contained: 1× PCR mix (GenLab, Moscow, Russia), 250 nM dNTP (Evrogen), 2 U of polymerase (GenLab), 300 nM primers and 200 nM short hydrolysis probes (Universal ProbeLibrary; Roche, Basel, Switzerland) or 1× TaqMan Gene Expression Assays (Thermo Fisher Scientific), and 1× LowROX (Evrogen). Quantitative PCR program was as follows: denaturation for 10 min at 95°C, 95°C for 15 s, and 60°C for 60 s for 50 cycles. All reactions were carried out in three technical replicates. TaqMan Gene Expression Assays representing primer–probe sets were used for reference genes: *RPN1*-Hs00161446_m1, *ACTB*-Hs01060665_g1. For the *HSPA1A* gene, we applied custom primers (forward: AAGGACCGAGCTCTTCTCG, reverse: GGTTCCCTGCTCTCTGTCG) and probe #47 from Universal ProbeLibrary (Roche). The RT-minus controls were used for each sample and each pair of primers. The expression level of the target gene under each condition was calculated using the qGEPS (ATG) tool (Melnikova et al., 2016) as follows:

$$\begin{array}{l} \text{Expression level}=2^{{\left(C_{t}^{\text{eff}}\right)}^{\text{reference gene}}-{\left(C_{t}^{\text{eff}}\right)}^{\text{target gene}}}\\ C_{t}^{\text{eff}}={\text{C}}_{\text{t}}\times \log_{2}\left(1+E\right) \end{array}$$

where *E* is reaction efficiency for each pair of primers (*E* = 1 corresponds to 100% efficiency); *Ct* is replicate-averaged threshold cycle. All reaction efficiencies were more than 90%.

### Mouse Model

Male Balb/c nu/nu mice aged 5 to 6 weeks were purchased at Shemyakin–Ovchinnikov Institute of Bioorganic Chemistry, Russian Academy of Sciences. All animal experiments were performed in accordance with European and Russian national guidelines for animal experimentation and were approved by the local animal and ethics review committee of the FSBSI “N.N. Blokhin NMRCO,” reference number 2017-010. A375 cells were implanted subcutaneously into the right flank of the mice to ensure successful solid tumor initiation and tumor growth measurements. A tumor volume was estimated by the following formula: *V* = (length × [width]^2^)/2. Experiments started when the tumor size reached 150 ± 20 mm^3^. Upconversion nanoparticles (100 μL, 2 mg/mL) were injected peritumorally. Tumor visualization and local overheating were demonstrated 2 h after administration. Near-infrared laser irradiation without UCNPs injection was used as a control.

## Data Availability Statement

The datasets generated for this study are available on request to the corresponding author.

## Ethics Statement

The animal study was reviewed and approved by Ethics review committee of the FSBSI N.N. Blokhin NMRCO, reference number 2017-010.

## Author Contributions

IK: development of a theoretical model. RA: providing *in vitro* experiment. VR: imaging system construction. DK and NS: small animal experiments. AN: UCNPs synthesis. NM and AD: qPCR analysis. AI: Yb3+ absorption data analysis. AG: UCNPs surface modification. EK: manuscript preparation, author of idea.

## Conflict of Interest

The authors declare that the research was conducted in the absence of any commercial or financial relationships that could be construed as a potential conflict of interest.
