# Supplementary material for: Local Overheating of Biotissue Labeled With Upconversion Nanoparticles Under Yb3+ Resonance Excitation
Source: Front Chem. 2020 May 8;8:295. doi: 10.3389/fchem.2020.00295 (PMC7225365; doi:10.3389/fchem.2020.00295)
Supplement: Supplementary file 1 [file Image_1.pdf]

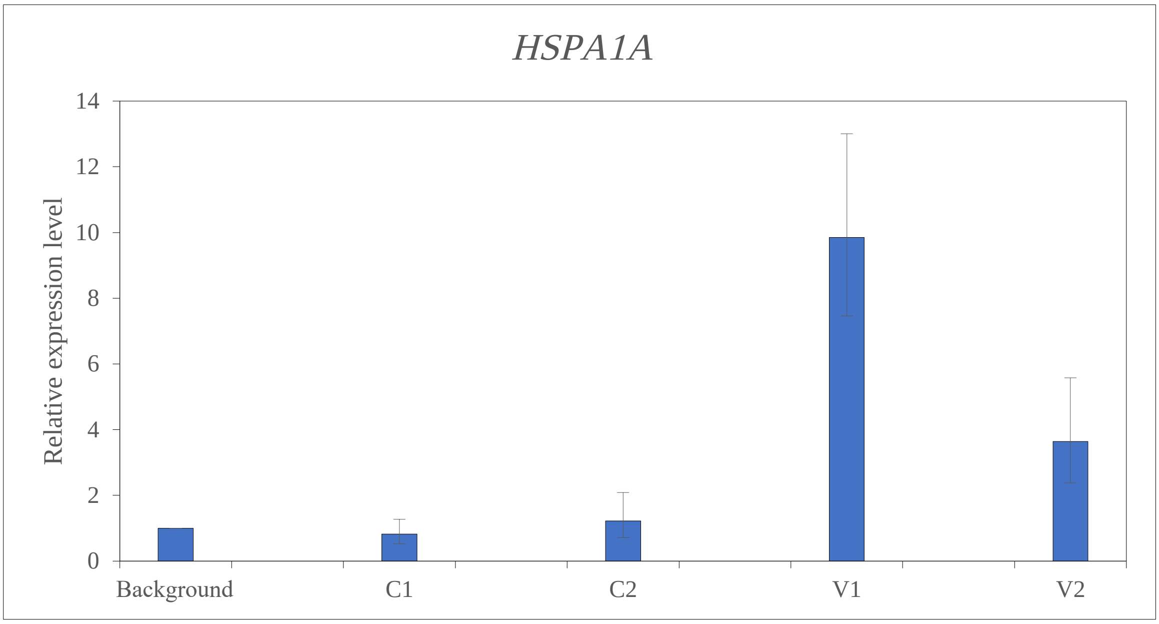

Supplementary data 1. The *HSPA1A* expression in human embryonic kidney HEK 293 cells, qPCR assay: background sample (no UCNPs, no NIR); control sample 1 (no UCNPs, NIR irradiation, T = 40.6±0.5 ^{0}C); control sample 2 (0.1 mg/mL UCNPs, no NIR); treated sample 1 (0.1 mg/mL UCNPs, NIR irradiation, T = 45 ^{0}C); treated sample 2 (0.1 mg/mL UCNPs, NIR irradiation, T = 40.6±0.5 ^{0}C). The *HSPA1A* expression in each sample was normalized to that in the background sample.
