# Supplementary material for: Local Overheating of Biotissue Labeled With Upconversion Nanoparticles Under Yb3+ Resonance Excitation
Source: Front Chem. 2020 May 8;8:295. doi: 10.3389/fchem.2020.00295 (PMC7225365; doi:10.3389/fchem.2020.00295)
Supplement: Supplementary file 2 [file Image_2.pdf]

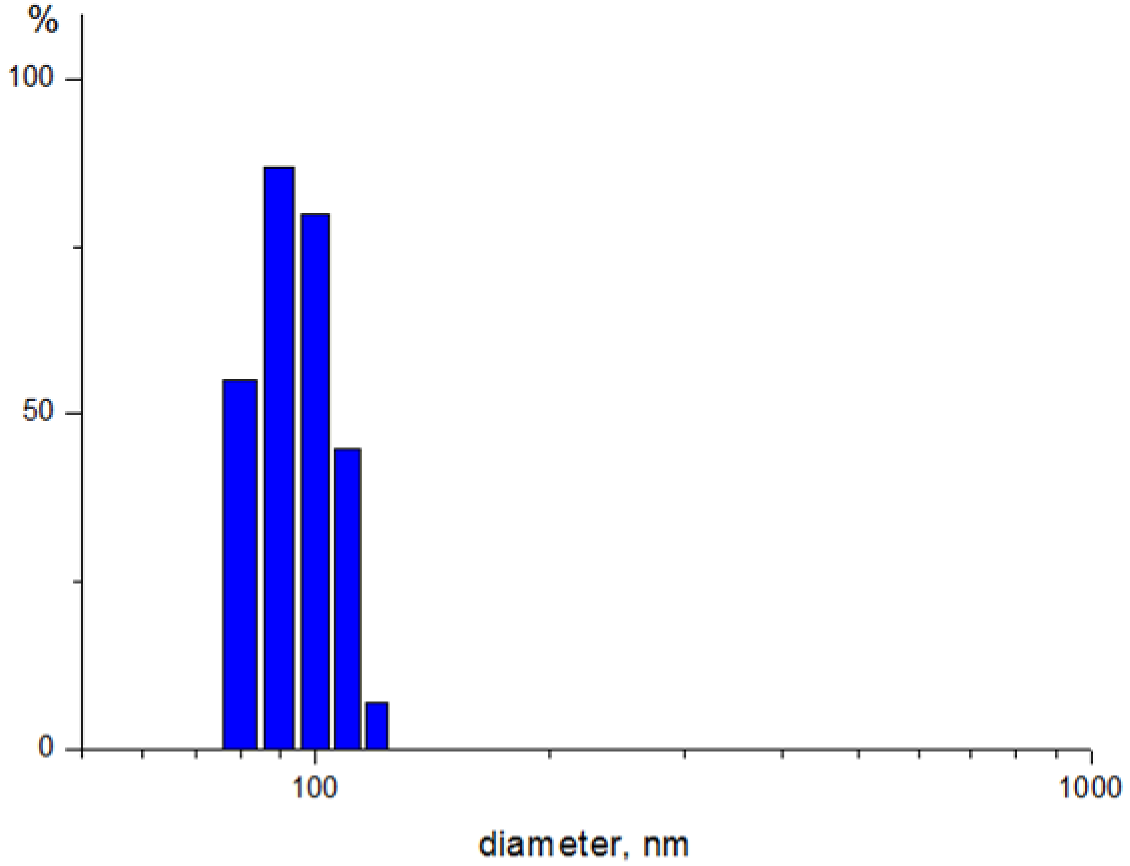

Supplementary data 2. The size distribution of UCNPs modified with PMAO-PEG shell (ζ = -41.1 mV) in the PBS buffer (pH 7.0).
